# Antiphospholipid syndrome and pregnancy—a hematologic perspective

**DOI:** 10.1016/j.rpth.2026.103396

**Published:** 2026-02-16

**Authors:** Andrew J. Doyle, Catrin Cox, Karen A. Breen

**Affiliations:** Haemostasis and Thrombosis Centre, Guy’s & St Thomas’ NHS Foundation Trust, London, UK

**Keywords:** abortion, habitual, antibodies, antiphospholipid, antiphospholipid syndrome, obstetric, placental insufficiency, preeclampsia, pregnancy complications, hematologic, thrombophilia

## Abstract

A state-of-the-art lecture entitled “APS and pregnancy: a haematologic perspective” was presented at the International Society on Thrombosis and Haemostasis (ISTH) Congress in 2025. Antiphospholipid syndrome (APS) is a multisystem autoimmune disorder associated with thrombotic and obstetric complications, which can often be difficult to diagnose and has limited treatment options. During pregnancy, APS presents a particularly complex set of issues with a high risk of thrombosis and adverse pregnancy-related outcomes. Since the initial description of APS by Graham Hughes et al in 1983, there have been many advancements in understanding of disease mechanisms, disease-defining criteria, and patient outcomes including pregnancy outcomes. However, there is still need for improvement, especially regarding risk stratification and optimal management to enhance pregnancy-related outcomes in APS. With few novel treatments are on the horizon, it is a clear reminder of the challenges involved in conducting clinical research during pregnancy. In this review, we will focus on a hematologic perspective of APS management relating to pregnancy, including discussion on prepregnancy counseling. There will be more of a focus on late adverse pregnancy outcomes and managing thrombosis-related issues and less emphasis on earlier pregnancy-related issues, such as recurrent miscarriage since this is more aligned with obstetric perspectives. Finally, we summarize relevant new data on this topic presented during the 2025 ISTH Congress.

## Essentials

• Antiphospholipid syndrome is a condition with increased risk of blood clots and pregnancy problems.
• Diagnosing antiphospholipid syndrome is often difficult.
• Management in pregnancy is with blood-thinning medicines and close and specialist monitoring.
• More research is needed to personalize treatment and improve outcomes.

## 1 Introduction

Antiphospholipid syndrome (APS) is a rare autoimmune condition causing a prothrombotic tendency due to autoimmune antibodies to β2-glycoprotein-1 and other phospholipid-binding proteins. APS has an incidence of ∼1 in 20,000 patients per year [1]. Manifestations include venous, arterial, or microvascular thrombosis known as thrombotic APS (TAPS), as well as adverse pregnancy outcomes (APOs), known as obstetric APS (OAPS). The most common types of thrombosis associated with APS are deep vein thrombosis, pulmonary embolism, and ischemic stroke [2]. Thrombosis recurrence is high in comparison with other thrombotic disorders despite anticoagulation, with vitamin K antagonists (VKAs) used as the mainstay of treatment. Recurrent early pregnancy loss (EPL) has been considered the most frequently reported APO of OAPS, while late pregnancy loss (LPL), pre-eclampsia (PEC) and intrauterine fetal growth restriction (IUFGR) related to placental insufficiency (PI) are recognized associations [3].

Making a diagnosis of APS can be challenging. It requires a detailed history, evaluation of other associated risk factors for thrombosis and APO, and consideration of factors that may affect laboratory test interpretation. For >20 years, the Sapporo and subsequent Sydney criteria have been the mainstay for classification of APS [4,5]. The more recent classification criteria produced by European Alliance of Associations for Rheumatology (EULAR)/American College of Rheumatology (ACR) have aimed to provide more clarity on defining cases of APS with a focus for homogenous cohorts, with a higher diagnostic specificity for research study inclusion [6]. Additionally, these criteria now include recognized nonthrombotic manifestations, such as thrombocytopenia and cardiac valve disease, albeit they are seen in varying degrees in clinical practice. The implications of these changes will be discussed further in this article.

During pregnancy, the use of low-molecular-weight heparin (LMWH) and aspirin have been the standard of care (SOC) for patients with both OAPS and/or TAPS with potential for adjunctive therapies, such as steroids, intravenous immunoglobulin (IVIg), hydroxychloroquine, and statins, to potentially improve outcomes. This review will focus on management during and after pregnancy from a hematologic perspective.

## 2 Pathogenic Mechanisms for Pregnancy-Related Complications in APS

Since the first description of APS in the early 1980s, research has better elucidated the disease features of the syndrome [7]. Distinct mechanisms have been identified potentially explaining differences between the obstetric features and thrombotic events seen in APS. It is important to remember that pregnancy itself causes its own distinct profile of hemostatic changes.

The presence of anti–β2-glycoprotein-1 antibodies have been recognized as a key initiating event in APS, in particular those targeting the D1 domain of the molecule [[8], [9], [10], [11]]. β2-Glycoprotein-1 is a ubiquitous phospholipid-binding protein that has a poorly defined physiological role but felt to be a key mediator between the coagulation and innate immune systems [2,12]. APL can bind to various cell types including platelets, neutrophils, endothelium, and trophoblasts via cell membrane receptors. The infusion of antiphospholipid antibodies (APLs) has been demonstrated to cause APO in healthy murine pregnancy models [[13], [14], [15]]. Despite the presence of APL, some patients do not develop clinical sequelae suggesting a ‘second hit’ is required that remains poorly defined.

Thromboinflammation is a key component in the pathogenesis of APS with activation of both the coagulation and innate immune system [16,17]. Analysis of patients and animal models demonstrate that the presence of APL leads to the activation of complement and secretion of various inflammatory chemokines such as tumor necrosis factor (TNF)-1, interleukin-6, and interleukin-8. Tissue factor is also secreted from monocytes, platelets, and the endothelium are activated with extracellular microparticle release, and there is neutrophil activation with NETosis [[18], [19], [20]]. The normal anticoagulant barrier to the endothelium, annexin A5, is also disrupted with the binding of APLs [21]. These may be potential therapeutic targets outside the setting of anticoagulation.

**Figure 1:**
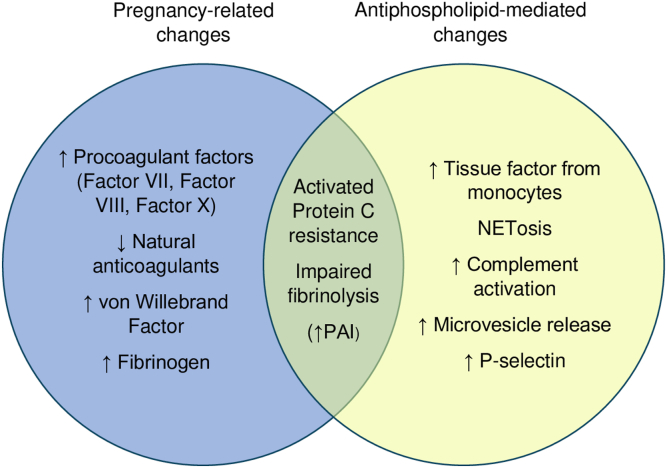
Hemostatic changes associated with pregnancy and antiphospholipid antibodies. NET, neutrophil extracellular trap; PAI, plasminogen activator inhibitor.

Thromboinflammatory changes caused by the presence of APL can lead to APO and thrombosis. The placenta in OAPS shows a combination of thrombotic and inflammatory features including placental infarction, impaired spiral artery remodeling, decidual inflammation, increased syncytial knots, and complement C4d deposition [22]. These features are not isolated to OAPS and can be seen in other conditions associated with PI [23]. Thrombotic risk is elevated in women with APS both in pregnancy and by the presence of APL with distinct prothrombotic mechanisms. Figure 1 illustrates these changes in coagulation and their potential overlap leading to hypercoagulability.

## 3 Diagnosing APS

In practice, confirming a diagnosis of APS can often be complex and usually requires a combination of clinical and laboratory features. Development of different diagnostic and classification criteria has led to further complexity for the clinician. The goal of developing a classification criteria has been to identify a set of patients with common characteristics for research purposes and a diagnostic criteria, rather than classification criteria, have been used in order to identify patients with the condition so appropriate treatment can be given. Classification and diagnostic criteria are often overlapping but classification criteria have often been more stringent in order to identify suitable patient cohorts appropriate for inclusion in research studies.

Classification of APS has changed over the last 40 years, with continued refinement of both clinical and laboratory features (Table 1). Initial classification criteria published in 1996 (the Sapporo criteria) focussed mainly upon clinical and laboratory definitions to classify APS, with all APO being equally weighted in significance [4]. Clinical criteria for OAPS include recurrent EPL (≥3 recurrent EPL of <10 weeks’ gestation), LPL, or preterm delivery due to PEC or PI (<34 weeks’ gestation).

**Table 1:** Changes in diagnostic and classification criteria of antiphospholipid syndrome over time.

	Sapporo (1999)	Sydney (2006)	EULAR/ACR (2023)
Clinical features			
Adverse pregnancy outcomes	RPL	RPL	Consecutive RPL
	Late pregnancy	Late pregnancy	Late pregnancy
	Pre-eclampsia	Pre-eclampsia	Pre-eclampsia
	IUFGR	IUFGR	Placental insufficiency
Impact of APO	Not weighted	Not weighted	Weighted with higher scoring for pre-eclampsia or placental insufficiency <34 wk^a^
Thromboses	Venous	Venous	—
	Arterial	Arterial	
	Microvasacular	Microvasacular	
Impact of thrombosis natural history	Not included	Not included	—
Laboratory features			
Timing between tests	>6 wk	>12 wk	>12 wk
Timing between test and clinical event	Not stipulated	Not stipulated	<3 y
Impact of testing	Not weighted	Not weighted	Weighted with higher scoring for persistent high-titer solid-phase assays (>80 IU/L) and lupus anticoagulant positive

ACR, American College of Rheumatology; APO, adverse pregnancy outcome; EULAR, European Alliance of Associations for Rheumatology; IUGFR, intrauterine fetal growth restriction; RPL, recurrent pregnancy loss.

a Defined as fetal weight <10th centile or postnatal birth weight of <10th centile for gestation age in absence of other causes and abnormal fetal-monitoring features or placental histological results.

PI-associated complications of early onset have a stronger association with APS. EPL is a common pregnancy-associated complication occurring in 30% to 40% of the general population with 1% to 5% of couples having recurrent EPL and not specific to a diagnosis APS [24,25]. In an aim to address some of these issues, EULAR/ACR working group published a new set of classification criteria in 2023 [6]. These criteria were established with a focus of creating a homogenous cohort of patients for clinical studies. For the first time, a weighted scoring system comprising both clinical and laboratory criteria have been included to improve the diagnosis of APS.

Clinical criteria for APO in EULAR/ACR 2023 criteria have remained largely the same although more detailed explanations are included to further classify histological placental changes suggestive of PI. Furthermore, more emphasis has been placed on presentations of PI and severe PEC, in comparison with recurrent EPL and LPL in the absence of PI.

In a further attempt to refine the thrombotic criteria for APS, weighting for those in higher-risk groups such as unprovoked venous thromboembolism (VTE) or stroke without cardiovascular risk factors have been added as well as further histological descriptions of microvascular thrombosis. Nonthrombotic clinical manifestations of valvular heart disease and thrombocytopenia although recognized associations of APL, were not included as part of clinical entities in the Sapporo and the subsequent Sydney classification criteria but are included as nonthrombotic clinical manifestations to increase the specificity of the condition [26,27].

The laboratory criteria for APS diagnosis include the persistent positivity for lupus anticoagulant (LA) IgM and IgG anti–β2-glycoprotein-1 or anticardiolipin antibodies on 2 occasions at least 12 weeks apart. The EULAR/ACR 2023 criteria continues to use persistent APL positivity although the time from clinical event to APL testing has been reduced to 3 years. Thresholds for low (<40 units), intermediate (>40-79 units), and high positivity (> 80 units) using ELISA have also been defined [28].

Interpreting APL testing profiles can be difficult in clinical practice. Patients with so-called low-titer–positive APLs—patients with results >99th centile of a normal population but <40 units—can be difficult to ascribe a diagnosis of APS [29]. There is conflicting data in regards to the relevance of these thresholds for APO and thrombosis risk [[30], [31], [32], [33]]. These low-titer results can be further compounded by using non-ELISA methods for detecting anticardiolipin and anti–β2-glycoprotein-1 antibodies that overestimate results in comparison to ELISA, which is the current gold standard [28,34,35]. The requirement for further work upon the standardization of APL testing platforms and the relevance of solid-phase antibody results was recognized in the International Society on Thrombosis and Haemostasis (ISTH) 2025 congress and in published guidelines and recommendations [36,37].

Furthermore, the correct timing of laboratory testing for APL is important. Testing should be done outside of an acute thrombotic episode and without the presence of anticoagulation, which affects the reliability of results, in particular LA. Clinicians should also be aware that LA testing during pregnancy may give either false-negative or false-positive results, so results outside of pregnancy should be used to guide treatment [36]. Noncriteria APL, in particular antiphosphatidylserine/antiprothrombin antibodies, have been recognized as potential pathogenic APL. Their inclusion into testing practice may also improve prognostication of the disease, although they are yet to be incorporated in wide-spread clinical practice and recent guidelines [38].

## 4 Outcomes of Pregnancies in APS

Pregnancy outcomes in women with APS can be divided into maternal- and fetal-related outcomes (Table 1). Complication rates are often much higher in APS than those seen in the women in the general population [39]. In a systematic review of studies assessing maternal outcomes, 770 women with APS were compared with 212,184 APL-negative controls. Rates of APO were significantly higher with APS compared to APL negative control pregnancies, in particular pregnancy-related thrombosis (relative risk [RR], 2.8), neonatal mortality (RR, 3.4), and preterm delivery (RR, 1.9) in APS pregnancies vs controls.

Interpreting studies on APO in APS can be challenging. Overall, there have been many limitations with studies performed to date. There is significant heterogeneity between patient groups included in published studies, including some patients not meeting criteria for APS, variable definitions of pregnancy outcomes, and most data being reported from retrospective studies with small cohort sizes [40]. Treatment regimens are varied, with some receiving aspirin alone or a combination of aspirin and heparin for varied durations for obstetric indications, and information on thrombotic complication rates is limited.

The APS ACTION (Antiphospholipid Syndrome Alliance for Clinical Trials and International Networking) registry commenced in 2010 with the aim of capturing standardized outcomes of patients with APS in the registry over time. Initial early data reported recurrent EPL in 27% of 55 APS pregnancies; 27% of the remaining 40 pregnancies progressing beyond the first trimester developed PI-related issues, such as IUGFR before 37 weeks’ gestation [3]. In addition, 87% had treatment with varying combinations of aspirin and prophylactic-dose LMWH.

Similarly, the Euro-Phospholipid Project, which also commenced in 2010, is a collaboration of over 30 hospitals, with the aim of collecting data on pregnancy outcomes in APS [41]. They reported recurrent EPL as the most common complication in an analysis of >1000 patient cases (retrospective and prospective) recorded in the registry. Like the APS ACTION registry, rates of PI-related complications remained high. It is important to note that only 45% received any of the recommended SOC treatment for OAPS. Of those who did, despite varied treatment regimes, 85% pregnancies had a successful live birth.

In pregnancies with APL, thrombosis rates of up to 12% have been reported in historic retrospective analysis; although contemporary, multicenter data on rates of thrombotic complications in pregnancy are limited [[42], [43], [44]]. In Table 2 [[45], [46], [47], [48], [49], [50], [51], [52], [53], [54]], we highlight the rates of VTE and stroke in the general population, APS and during pregnancy with APS. Although not direct comparisons, we feel this highlights the thrombotic risk that these women face during pregnancy. Recurrent thrombosis is reported as mainly recurrent VTE as being the most common thrombosis in the general population. However, this may not include arterial events, such as stroke, which are common in APS. Catastrophic APS (CAPS) is a much less frequent manifestation and occurs in <1% of APS pregnancies. Pregnancy itself is a recognized trigger for CAPS, occurring mainly in the postpartum period, with high rates of associated mortality [55].

**Table 2:** Comparative rates of thrombotic events during pregnancy, antiphospholipid syndrome, and the general population

Thrombotic event	General population (% events/y)	Younger patients (% events/y)^a^	Recurrence in TAPS (% events/y)	Pregnancy (% events/pregnancy)	Pregnancy with APS (% events/pregnancy)
VTE	0.1 [45]	<0.002 [45]	2-5 [46]	0.001-0.1 [45,47]	1 [48]
Stroke	0.1 [49]	<0.05 [50]	5-10 [51,52]	<0.001 [53]	11^b^ [54]

APS, antiphospholipid syndrome; TAPS, thrombotic antiphospholipid syndrome; VTE, venous thromboembolism.

a Younger than 60 y.

b With a history of previous ischemic stroke.

## 5 Risk Stratification for Pregnancy with APS

To improve pregnancy-related outcomes in APS, there is a need for the following: (1) to identify those at thrombotic risk, (2) to identify those at risk of APO, and (3) to identify those at high risk of anticoagulation-related bleeding during pregnancy. Disease phenotype and antiphospholipid testing patterns can provide some guidance to the clinician.

A clinical history has been shown to predict future pregnancy outcomes. For example, women with a history of thrombosis compared with those with APO alone have a higher risk of preterm delivery, IUGFR, and further thrombotic outcomes (odds ratio, 6.26 to APL-negative controls) [56]. Interestingly, women who have presented with recurrent EPL alone seem to have reduced risk of second and third trimester APO [57]. In terms of APL testing patterns, women who are positive for LA and those who are triple positive have been shown to have worse outcomes. This feature appears to apply for both risk of pregnancy-related thrombosis and APO [56,58]. Women with a concomitant connective tissue disorder have also been shown to have worse pregnancy outcomes [59].

Attempts have been made to risk stratify patients with APS outside of pregnancy. Scoring systems, such as Global Antiphospholipid Syndrome Score (GAPSS) and the adjusted GAPSS using APL testing pattern and cardiovascular risk factors, have been developed in attempt to help predict thrombotic outcomes using a retrospective review of variables [60,61]. These have not yet been properly validated, particularly in pregnancy in a prospective setting, but there appears to be some utility in using APL pattern for high-risk pregnancies based on retrospective cohort data [62,63]. For example, those with very high GAPSS had low rates of live births at ∼40% despite SOC treatment.

Personalized medicine may hold a key in the future when it comes to risk stratification in APS, and there is likely to be a rich development of risk-prediction tools with the advent of artificial intelligence. Models such as the EUREKA algorithm have already been shown to potentially help predict obstetric outcomes; however, these are again limited by retrospective data and small cohort sizes [64]. After developing better assessment tools for high-risk women with APS with data from international standardized registries, we can then aim to optimize treatment intensities and approaches to improve pregnancy outcomes.

## 6 Treatment Options During Pregnancy with APS

The mainstay of treatment during pregnancy is a combination of aspirin and LMWH, tailored to the individual’s medical history including preceding obstetric and thrombotic-related complications. Adjunct therapies may also be considered in those with refractory APS, that is, previous APOs despite SOC treatment.

Most trials in APS-related pregnancies have been focused on improving outcomes of recurrent miscarriage (RM) and have mainly compared use of aspirin alone with that of a combination of aspirin and heparin [56]. Many systematic reviews of these trials have similarly concluded that it is difficult to draw any firm conclusions given limitations in earlier studies as discussed in the previous section. In 2024, the 16th International Taskforce on OAPS concluded, “[the] collection of appropriate control populations in ongoing, well-structured, prospective, observational studies and registries are likely to be critical to successful future treatment” [65].

Recent prospective studies in pregnancy with a focus of thrombosis or APS have struggled with slow recruitment, competing patient priorities, underprioritization from funders, and overregulation or risk aversion by regulatory bodies and pharmaceutical companies [66,67]. At present, the HYPATIA study, a double-arm, blinded, randomized controlled study comparing the addition of hydroxychloroquine in addition to the SOC in women with persistent APLs, has faced many of these challenges [68].

Guideline recommendations from EULAR, British Society of Haematology, Royal College of Obstetricians and Gynaecologists, ACR, and American College of Chest Physicians for management of women with APS in pregnancy are outlined in Table 3 [36,37,[69], [70], [71], [72], [73]]. Broadly speaking, recommendations are considered according to patient groups: women with OAPS associated with early pregnancy complications (recurrent EPL), women with OAPS with later complications including LPL and PI-related complications, and refractory OAPS. Women with TAPS are considered separately as are women with noncriteria APS or isolated APL.

**Table 3:** Recommendations for the management of obstetric and thrombotic antiphospholipid syndrome according to international guidelines

Guideline	OAPS: recurrent EPL	OAPS: PI/LPL	TAPS	Incidental APL or noncriteria for OAPS: <3 EPL	Refractory OAPS
EULAR [37]	Prophylactic LMWH and aspirin	Prophylactic LMWH and aspirin	Treatment-dose LMWH and aspirin	Low-dose aspirin (75-100 mg daily)	Consider steroids or IVIg
BSH [36]	Prophylactic LMWH and aspirin	Prophylactic LMWH and aspirin	Treatment-dose LMWH and aspirin	Low-dose aspirin (75-100 mg daily)	Consider HCQ
RCOG [70,71]	Prophylactic LMWH and aspirin	Prophylactic LMWH and aspirin	Treatment-dose LMWH and aspirin	Low-dose aspirin (75-100 mg daily)	—
ACCP [72]	Prophylactic LMWH and aspirin	Prophylactic LMWH and aspirin	Treatment-dose LMWH and aspirin	Low-dose aspirin (75-100 mg daily)	—
ACR [73]	Prophylactic LMWH or aspirin	NA	Treatment-dose LMWH and aspirin	Low-dose aspirin (75-100 mg daily)	Consider HCQ

ACR, American College of Rheumatology; ACCP, American College of Chest Physicians; APL, antiphospholipid antibody; BSH, British Society of Haematology; EPL, early pregnancy loss; EULAR, European Alliance of Associations for Rheumatology; HCQ, hydroxychloroquine; IVIg, intravenous immunoglobulin; LMWH, low-molecular-weight heparin; LPL, late pregnancy loss; OAPS, obstetric antiphospholipid syndrome; PI, placental insufficiency; RCOG, Royal College of Obstetricians and Gynaecologists; TAPS, thrombotic antiphospholipid syndrome.

Women with a history of thrombosis with persistent APL require anticoagulation during pregnancy and in the postpartum period. Women taking warfarin or other anticoagulants should be counseled on the associated risk of fetal developmental issues and advised to switch to LMWH on confirmation of a positive pregnancy test. There is no clear evidence to suggest that changing to LMWH in the prepregnancy period reduces this risk in APS and other thrombotic conditions. Current guidelines recommend use of LMWH in combination with aspirin. Treatment-dose LMWH, rather than prophylactic dose, is recommended in most current guidelines in those with a history of TAPS regardless of the nature of the thrombotic history [36,37]. This varies from the approach in non-APS pregnancies with a history of VTE, where thromboprophylactic doses are used. Women with a history of TAPS can have varying thrombotic complications from those with a history of arterial thrombosis such as stroke, which are considered a higher risk cohort to those with a deep vein thrombosis in association with a major transient risk factor not usually on anticoagulation outside of pregnancy.

There is no evidence of the benefit of routine anti-Xa monitoring of LMWH in APS except for women at the extremes of body weights or with significant renal impairment (ie, creatinine clearance < 30 mL/min).

### 6.1 Management of acute thrombosis in pregnancy

In women with an acute thrombotic episode during pregnancy, it is recommended that initial treatment to stabilize the patient should be similar to those without APS [36]. Management will often include a dose escalation of anticoagulation to therapeutic intensity. Assessment for additional interventional therapies may be considered, such as thromboaspiration for high- and intermediate high–risk pulmonary embolism and acute ischemic stroke [[74], [75], [76]]. However, joint decision making should be made between obstetricians, obstetric physicians, intensivists, hematologists with expertise in APS, and other relevant specialities to assess hemodynamic instability, therapeutic intent, and bleeding risk. Following stabilization of the acute event, we suggest the continuation of treatment-dose LMWH until the postpartum with consideration for long-term oral anticoagulation. VKA can be considered safe while breastfeeding.

Although rare, CAPS can be a life-threatening presentation in pregnancy with both rapid development of macrovascular and microvascular thromboses. In addition to anticoagulation, treatment with therapeutic plasma exchange and immunosuppressive therapies, such as high-dose steroids, rituximab, and/or eculizumab, may need consideration [36]. It is important to recognize that CAPS can often be confused with severe presentations of HELLP (hemolysis [H], elevated liver enzymes [EL], and low platelets [LP]) syndrome, which usually resolves on delivery in contrast to CAPS that may often show progression until appropriate anticoagulation and immunosuppression are given.

### 6.2 Adjunctive therapies

Although aspirin and LMWH appear to be effective in many women with APS during pregnancy, there are those who need alternative or additional treatments. Options for consideration in pregnancy including steroids, IVIg, and hydroxychloroquine will be discussed in the next section. There is also the consideration that giving these options earlier to those considered at potentially high risk of APO may reduce these rates further as is the topic of clinical research [68].

### 6.3 Role of multidisciplinary care

Combined obstetric and hematology care is crucial for management of these patients. Close obstetric monitoring throughout pregnancy with carefully planned delivery is a critical component in ensuring good outcomes for these patients with early support from specialist midwives. Further advice may be needed from other medical specialists if there are other relevant diagnoses or organ injury from previous thrombosis, such as rheumatology, respiratory medicine, and cardiology. Additional monitoring including use of uterine artery Doppler at 20 weeks have been shown to predict poor outcomes, which helps guide regular growth scan monitoring and/or planning for earlier delivery as necessary [77,78].

## 7 Practical Issues often not Addressed in the Guidelines

In practice, clinicians are regularly faced with many additional diagnostic and treatment challenges when managing women with APS during pregnancy. In this section, we aim to address some of the challenges commonly faced by hematologists in the clinic setting.

### 7.1 Aspirin—what is the most appropriate dose and when to stop?

Currently, most guideline recommendations advise a dose of aspirin 75 mg daily. There is evidence that in some women a higher dose of aspirin 150 mg should be considered particularly those of increased body weight, a history of PEC, and age of <35 years [79]. PI-related complications associated with APL are usually experienced before 34 weeks’ gestation, and therefore, it is common practice to advise stopping aspirin by 36-38 weeks gestation [80].

### 7.2 What dose of LMWH should be given?

Guidelines advise to use either prophylactic or treatment-dose LMWH based upon the presence or absence of a thrombotic history. For those women with a thrombotic history with APS, most guidelines suggest treatment-dose LMWH but do not take bleeding risk into consideration. In the High-low study, women with a history of previous VTE were randomized to either prophylactic or intermediate-dose LMWH during their current pregnancy. The latter did not confer additional protection overall for VTE in pregnancy, although it was not associated with increased bleeding risk compared with prophylactic-dose LMWH [81]. However, <5% had a known thrombophilia, raising the applicability of these dosing to APS.

Similar bleeding rates are seen in large registries of pregnant women with APS reporting 2% to 3% major bleeding rates albeit with poor reporting of treatment intensity and with most bleeding events occurring in patients on nontherapeutic-dose anticoagulation and with concurrent aspirin use. Bleeding complications in APS in general have been related high-intensity anticoagulation, the use of concurrent antiplatelets, presence of thrombocytopenia, and other autoimmune conditions [[82], [83], [84]].

Consideration should be given to LMWH regimes based on the balance of thrombotic risk, bleeding, and treatment burden during pregnancy with APS [85]. We feel that features such as the timing of the last thrombotic event in relation to pregnancy, arterial vs venous events, and any associated provoking factors should be considered in decision making on the need for dose escalation of LMWH.

Careful planning and consideration of bleeding risk is required for any invasive procedures during pregnancy, delivery, and the puerperium, including neuraxial analgesia and fetal cell sampling. Emergency cesarean section has been reported as a key risk factor for bleeding with anticoagulation in previous APS–pregnancy cohorts. Additionally, no neuraxial bleeding events were reported if anticoagulation was ceased >12 hours prior to delivery in this cohort [67,85]. However, we recommend that >24 hours should elapse after therapeutic-intensity LMWH in keeping with current guidelines.

### 7.3 Can assisted reproductive therapy be consider with APS?

There is limited guidance for women with APL undergoing assisted reproductive therapy, including intracytoplasmic sperm injection [73]. Pregnancy loss has been demonstrated to be higher in women with APS than that in normal populations, but this can be reduced with pre-emptive SOC treatment [86,87]. The presence of APL is not thought to be associated with implantation failure with similar pregnancy rates overall [87]. We suggest careful planning and standard management of women according to how they are categorized in terms of prior complications. Careful planning may be required for any assisted reproductive therapy–related procedures for those with TAPS, including consideration of risk for any hormonal stimulation that may cause thrombosis and anticoagulation bridging for egg retrieval and embryo transfer to reduce bleeding risk.

### 7.4 Refractory obstetric APS

In those women who experience APO despite SOC treatment with aspirin and LMWH, adjunctive therapies may be considered [37]. These include steroids, hydroxychloroquine, statins, IVIg, and newer options, such as TNF-1 inhibitors, which have shown some potential benefit in previous studies [37,73,[88], [89], [90], [91], [92]]. There is no evidence for dose escalation of LMWH in women with refractory OAPS. However, the role of these adjunctive therapies is yet to be established, and while tempting to consider patients who have clearly had previous poor obstetric outcomes, we await outcomes of ongoing clinical trials, such as those assessing hydroxychloroquine and certolizumab before advocating the use.

### 7.5 Seronegative and noncriteria OAPS

Managing women who do not meet criteria for APS is a relatively common scenario presented to clinicians and is often difficult to navigate. This includes patients who may have been labeled as having seronegative APS on the basis of strong clinical suspicion but in whom screening for APL testing remains negative [93]. Patients diagnosed with seronegative APS typically have a diagnosis based on clinical symptoms or signs, particularly if there is a history of both thrombosis and APO. It is difficult to offer the same recommendations as those with a confirmed laboratory diagnosis and to advise management with LMWH and/or aspirin is only considered based on relevant clinical thrombotic and obstetric history, such as PEC [94]. This is particularly relevant in women with recurrent EPL alone; the ALIFE study showed no benefit with the additional of aspirin or aspirin and nadroparin over placebo alone, demonstrating subsequent live birth rates of between 61% and 69% regardless of treatment group [95].

A similar cohort include those labeled with noncriteria APS—such as women with non-RM with <3 EPLs and those with low-titer APL, who, in some publications, have been reported to have similar poor outcomes to those with higher titers [32]. In time, new research may help to improve provide answers on the association with APL. But, for now, most of these women should be considered as having incidental APL and managed as such.

### 7.6 Management of the postpartum period and after pregnancy loss

VKAs and LMWH are safe for use in women who are breastfeeding; therefore, for those on long-term anticoagulation, switching back to warfarin or alternative VKA is appropriate. For women not on long-term anticoagulation, it is recommended to continue LMWH for 6 weeks in the postpartum period for those deemed at high risk and for 10 to 14 days for those considered low or intermediate risk for thrombosis, which we discuss further [37,70].

Similarly, for those women experiencing pregnancy loss—either EPL or LPL—no guidance exists on the duration of thromboprophylaxis for those not on long-term anticoagulation. We suggest continuing LMWH thromboprophylaxis for up to 2 weeks following an EPL or for up to 6 weeks for third trimester loss, particularly if close to the expected delivery date. These should be taken into other risk factors at the time, including maternal age, smoking status, and obesity [70].

## 8 How to Approach Prepregnancy Counseling for Women with APS

**Figure 2:**
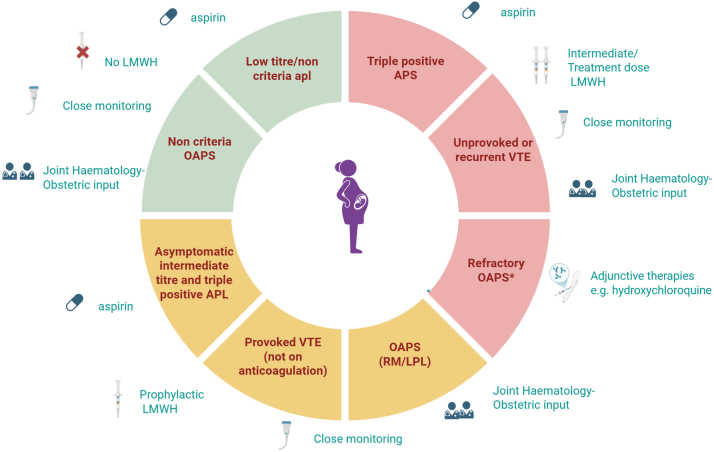
Approach to prepregnancy counseling in antiphospholipid syndrome (APS) during pregnancy. Green segments show low-risk groups not requiring low-molecular-weight heparin (LMWH). Yellow segments show intermediate-risk groups requiring LMWH. Red segments show high-risk groups requiring treatment escalation with either higher intensity LMWH or adjunctive therapies. ∗Prophylactic dose LMWH should be used in refractory obstetric antiphospholipid syndrome (OAPS). Created with Biorender. APL, antiphospholipid antibody; APS, antiphospholipid syndrome; LPL, late pregnancy loss; RM, recurrent miscarriage; VTE, venous thromboembolism.

Broadly speaking, we consider women as at low, intermediate, or high risk according to their clinical history and APL profile for the purposes of prepregnancy counseling—we have aimed to capture this approach in Figure 2. This is based upon authors’ experience and extrapolation of the available evidence and guidelines discussed in the previous sections of this article. Women should be considered on a case-by-case basis, and the balance of treatment should always be weighed against clinical risk, which includes considering both maternal and fetal APL-related outcomes.

We consider a low-risk profile to include women who do not meet criteria for a diagnosis of APS, that is, those with asymptomatic low-titer APL or noncriteria APS with <3 RM. The authors suggest that until further research or guidance is given, these low-titer results should be interpreted with caution and other contributing factors should be considered. Aspirin alone is likely to be most appropriate in this setting or inclusion into clinical studies.

The intermediate-risk group includes women with OAPS (RM and LPL), women with asymptomatic triple-positive APL, and those with medium-/high-titer APL (not triple positive) and previous provoked VTE (not on long-term anticoagulation). For this cohort of patients, the use of aspirin and prophylactic-dose LWMH is suggested during pregnancy. Going forward, however, it may also be appropriate to consider women with RM as a low-risk cohort and consider aspirin alone as sufficient.

Finally, the high-risk group include women with TAPS who are on long-term anticoagulation due to previous unprovoked VTE or arterial thromboembolic event including those with triple-positive APS, and those with continuing APO despite SOC treatment. Currently, guidelines for TAPS suggest use of treatment-dose LMWH in this cohort, but there are no data to support this practice. We suggest that the use of intermediate-dose anticoagulation may be considered in women with TAPS (nonacute) in order to reduce bleeding risk but offer sufficient cover to prevent recurrent thrombosis during pregnancy. Women who have had previous APO despite optimal management should also be considered as a higher-risk cohort but requiring a different treatment approach. In addition to the use of both aspirin and LMWH (prophylactic dose for the OAPS group), we recommend consideration of adjunctive treatments as appropriate although the preferred therapy is yet to be established. We encourage the enrolment into clinical trials for adjuvant treatments for women with refractory OAPS who fall within this group. Attention should be paid to other risk factors including stopping smoking, controlling hypertension and elevated cholesterol.

## 9 ISTH Congress Report

The ISTH Congress 2025 held in Washington provided various sessions related to women’s health and APS. There was a recognition that optimal management of OAPS and TAPS in pregnancy remains difficulty with a lack of robust, up-to-date data to support current management strategies.

There was discussion regarding the variability in results for different testing platforms for semiquantitative anticardiolipin and anti–β2-glycoprotein-1 antibodies, with higher equivalent levels for chemiluminescence assays than ELISA, which may cause difficulty in ruling out APS in those with the presence of low-titer results. There is ongoing work within the APS Scientific Subcommittee to standardize testing [34].

Additionally, there was controversy in the use of EULAR–ACR classification criteria for clinical studies described by Oviedo et al. and its application of those diagnosed clinically by the revised Sapporo criteria, which has been similarly described by other groups [[96], [97], [98]]. There was a particular concern that >80% of those with OAPS, especially those with recurrent EPL, diagnosed by the revised Sapporo criteria would, now, not be recognized the EULAR–ACR definitions and would not be suitable if used as an inclusion criterion for clinical studies. Subsequently, this raises the applicability of using a stricter classification criterion to clinical practice [99]. Future studies in OAPS will have to address this quandary.

Finally, Branch et al. [92] discussed the APO in OAPS with relation to the adjunct use of novel therapies to the backbone of LMWH and aspirin. Findings were presented from the IMPACT study assessing the outcomes of a single-arm, open-label study using certolizumab pegol, an anti–TNF-1 monoclonal antibody that has little placental transfer due to the absence of an Fc domain [92]. Certolizumab was given subcutaneously fortnightly at a loading dose of 400 mg for 3 doses and then subsequently 200 mg until 28 weeks’ gestation. The study end point report was a composite of late pregnancy loss of ≥10 weeks, severe PEC, or PI, leading to delivery at <34 weeks, but did not include EPL. They demonstrated that the composite outcome of these adverse pregnancy outcomes was 20% in a cohort of 45 women with comparison with a historical well-defined control group from the PROMISSE study of 37% treated with LMWH and aspirin, offering hope to those with refractory OAPS [100].

## 10 Future Directions for APS in Pregnancy

It remains clear that there is an unmet need for managing women with APS in pregnancy. Many questions remain around optimal treatment with aspirin and LMWH including dosing, duration, and the role for adjunctive therapies. We await outcomes from currently enrolling trials such as HYPATIA with anticipation. Risk profiling will be key to a more personalized approach to counseling and management of pregnancy in APS.

Research in obstetric medicine, not least in the setting of APS is difficult, due to large cohorts required to provide comparative data between different patient groups or therapeutics of interest and an overrepresentation of specialist centers that may treat more refractory or complicated patients. Multicenter, international collaboration seems the most appropriate approach moving forward.
